# Severity of Sjögren’s Syndrome Keratoconjunctivitis Sicca Increases with Increased Percentage of Conjunctival Antigen-Presenting Cells

**DOI:** 10.3390/ijms19092760

**Published:** 2018-09-14

**Authors:** Stephen C. Pflugfelder, Fang Bian, Koray Gumus, William Farley, Michael E. Stern, Cintia S. De Paiva

**Affiliations:** 1 Department of Ophthalmology, Baylor College of Medicine, Houston, TX 77030, USA; Fang.Bian@bcm.edu (F.B.); dkorayg@hotmail.com (K.G.); Bill.Farley@bcm.edu (W.F.); michaelestern4@gmail.com (M.E.S.); cintiadp@bcm.edu (C.S.D.P.); 2 Department of Ophthalmology, Erciyes University School of Medicine, 38039 Kayseri, Turkey

**Keywords:** Sjögren’s syndrome, dry eye, goblet cells, antigen-presenting cells, dendritic cells

## Abstract

This study investigated the relationship between clinical severity and percentage of conjunctival antigen-presenting cells (APCs) in Sjögren’s syndrome (SS)-associated keratoconjunctivitis sicca (KCS). KCS clinical severity was based on symptom severity, tear volume, tear break-up time, and ocular surface dye staining. Conjunctival goblet cell density (GCD) was measured in periodic acid Schiff (PAS)-stained membranes. Conjunctival cells obtained by impression cytology were used for flow cytometry to measure percentages of CD45^+^HLA-DR^+^ APCs and mature CD11c^+^CD86^+^ dendritic cells (DCs). Compared to normal conjunctiva, the percentages of HLA-DR^+^ and CD11c^+^CD86^+^ cells were higher in the conjunctiva of the KCS group (*p* < 0.05). The percentage of CD45^+^HLA-DR^+^ cells positively correlated with clinical severity (*r* = 0.71, *p* < 0.05) and negatively correlated with GCD (*r* = −0.61, *p* < 0.05). Clinical severity also negatively correlated with GCD (*r* = −0.54, *p* < 0.05). These findings indicate that a higher percentage of APCs and mature DCs in the conjunctiva is associated with more severe KCS in SS. These APCs may contribute to the generation of the pathogenic Th1 cells that cause goblet cell loss in KCS.

## 1. Introduction

Sjögren’s syndrome (SS) causes severe aqueous-deficient dry eye and ocular surface disease, termed keratoconjunctivitis sicca (KCS) [1,2]. Dysfunction and loss of mucin-producing conjunctival goblet cells is a key pathological feature of SS KCS [1,3]. Increased expression of the T helper 1 (Th1) cytokine Interferon gamma (IFN-γ), which induces an unfolded protein response and apoptosis in (goblet cells, has been found in KCS [3,4,5]. In addition to producing mucins that lubricate and protect the ocular surface, goblet cells have also been found to produce immunomodulatory factors, including retinoic acid, transforming growth factor beta 2 (TGF-β2), and mucin 2 (MUC2), which are important for maintaining immune tolerance [6,7,8,9,10]. Conjunctival goblet cells serve as passages for ocular surface antigens to antigen-presenting cells (APCs) located in the basement membrane zone and stroma [6]. Goblet cell mucin has been found to mix with ovalbumin (OVA) antigen as it passes through GAPs, and immune tolerance to topically applied OVA is lost in the sterile alpha motif (SAM)-pointed domain containing (E-twenty-six) ETS transcription factor (Spdef) knockout (KO) mouse strain that lacks goblet cells [6,8]. Goblet-cell-produced retinoic acid has been found to suppress differentiation and stimulate production of the Th1-inducing cytokine interleukin 12 (IL-12) by bone-marrow-derived APCs [9]. The Spdef strain has been found to have a higher number of IL-12-producing APCs in the conjunctiva compared to the wild-type strain [8].

The percentage of HLA-DR^+^ cells in the conjunctiva has been reported to increase in dry eye and has been used as a severity marker [11]. HLA-DR is primarily expressed by APCs, including monocytes, macrophages and dendritic cells, but it can be induced in epithelial cells that are exposed to inflammatory cytokines, such as IFN-γ [12]. Previously reported studies have compared the percentages of HLA-DR^+^ cells between normal and dry eyes in the entire conjunctival cell population or in epithelial cells alone [13,14,15,16,17]. Changes in the percentages of APCs and the relationship between antigen-presenting and goblet cell densities in SS KCS has not been investigated. The purpose of this study was to investigate the relationship between clinical severity and density of conjunctival APCs and goblet cells in SS-associated KCS.

## 2. Results

### 2.1. Clinical Severity 

**Figure 1:**
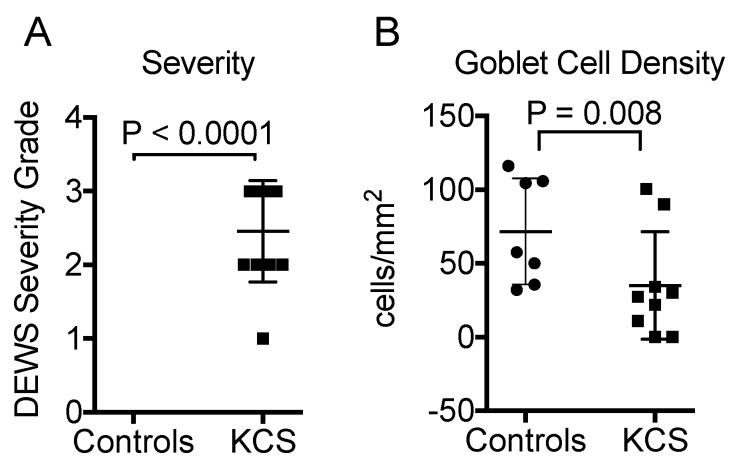
Clinical severity (**A**) and conjunctival goblet cell density (**B**) in controls (*n* = 7) and Sjögren’s syndrome keratoconjunctivitis sicca (KCS) subjects (*n* = 9). Mean + standard deviation (SD). Mann–Whitney test.

Eight control subjects and 11 SS KCS patients were enrolled. Goblet cells could not be evaluated in samples from one control and two SS patients because of poor sample quality, and they were excluded from all statistical analyses. The control subjects had no eye irritation symptoms and no clinical signs of KCS. The KCS patients had a mean Dry Eye Workshop (DEWS) clinical severity score of 2.5 ± 0.72 (range 1–3; Figure 1). The goblet cell density (GCD) was 42% lower in the KCS group (39.6 ± 40.5) than the control group (68.2 ± 40.4, *p* = 0.008 vs. KCS; Figure 1).

### 2.2. Conjunctival Antigen-Presenting Cells

The percentage of bone-marrow-derived CD45^+^ cells in the conjunctiva obtained by impression cytology from all three sites—superior bulbar (SB), nasal bulbar (NB), and temporal bulbar (TB)—was significantly higher in the KCS group (*p* < 0.02) and approached significance in cells obtained from the SB alone (*p* < 0.06).

**Figure 2:**
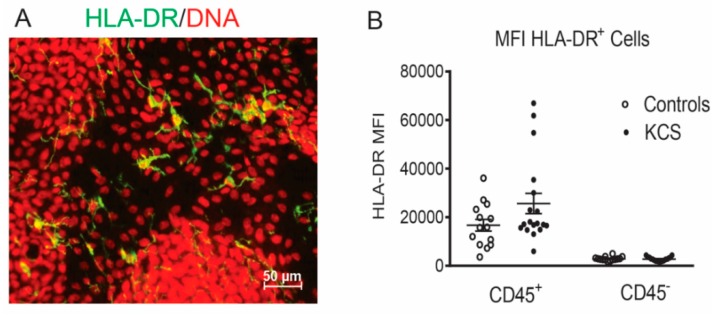
HLA-DR intensity in gated CD45^+^ cells. (**A**) Merged image of laser confocal micrograph of dendritic cells (DC) in human conjunctival impression cytology from a normal subject stained with HLA-DR (green) and nuclei stained with red propidium iodide. (**B**) Mean fluorescent intensity (MFI) of HLA-DR^+^ cells in CD45-gated cell population obtained by conjunctival impression cytology from controls (*n* = 7) and KCS subjects (*n* = 9). Data shown as scatter plots with mean ± SD.

**Figure 3:**
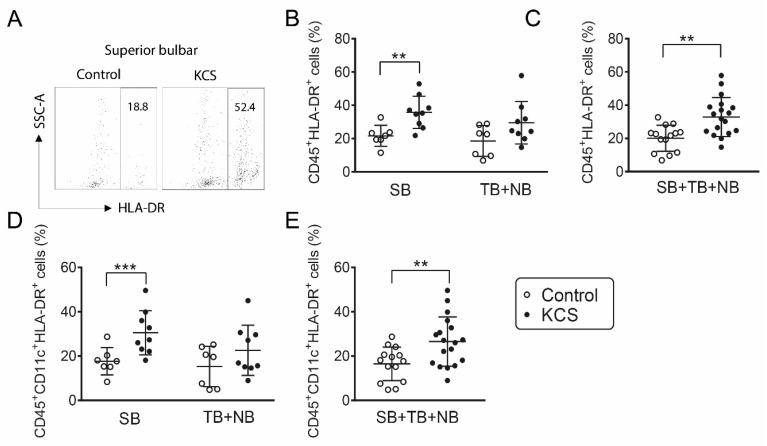
Increased percentage of HLA-DR^+^ cells in KCS patients. (**A**) Representative dot plots of flow cytometry showing HLA-DR^+^ cells gated from live CD45^+^ population in the control and KCS samples. (**B**) Accumulative data showing percentage of CD45^+^HLA-DR^+^ cells in the superior bulbar (SB) and nasal bulbar (NB) + temporal bulbar (TB) conjunctiva in the control (*n* = 7) and Sjögren’s syndrome (SS) subjects (*n* = 9) and (**C**) the corresponding accumulative data for all three bulbar conjunctival sites. (**D**) Accumulative data showing percentage of CD45^+^CD11c^+^HLA-DR^+^ cells in SB and NB + TB conjunctiva in the control (*n* = 7) and SS subjects (*n* = 9) and (**E**) the corresponding accumulative data for all three bulbar conjunctival sites. Data shown as scatter plots with mean ± SD. ** *p* < 0.01; *** *p* < 0.001, Mann–Whitney test.

**Figure 4:**
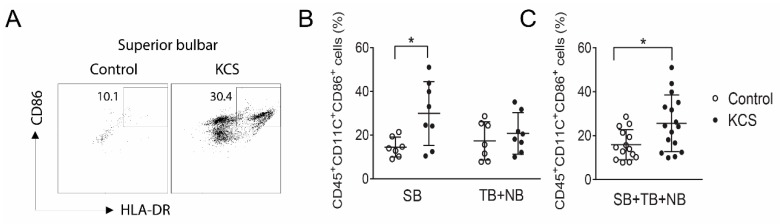
(**A**) Representative scatter plots of HLA-DR^+^CD86^+^ in representative control and KCS patient. (**B**) Graphs comparing percentage of CD11c^+^CD86^+^ cells in the SB and NB + TB conjunctiva in the control (*n* = 7) and SS subjects (*n* = 8) and (**C**) corresponding graphs for all three bulbar conjunctival sites. Data shown as scatter plots with mean ± SD. * *p* < 0.05 Mann–Whitney test.

The percentage of HLA-DR^+^ cells obtained by conjunctival impression cytology from dry eye subjects has been found to be significantly higher than the control in a number of previously reported studies [13,14,15,17]. HLA-DR is expressed by APCs but can also be expressed by epithelial cells that are exposed to inflammatory/immune cytokines, such as IFN-γ [12]. We examined differences in bone-marrow-derived HLA-DR^+^ cells between control and SS KCS because these cells can present antigen and initiate adaptive immunity. HLA-DR^+^ antigen-presenting cells have been noted in and below the conjunctival epithelium in humans and mice (Figure 2A) [6,18]. As confirmation of our gating strategy on CD45^+^ cells, we found that >99% of the HLA-DR mean fluorescence intensity (MFI) was in CD45^+^ cells obtained from the conjunctiva of the control and KCS eyes (Figure 2). The percentages of HLA-DR^+^CD45^+^ antigen-presenting cells and HLA-DR^+^CD45^+^CD11c^+^ dendritic cells were higher in the SB and the combined (SB + NB + TB) groups in KCS than the control eyes (Figure 3A–E); however, percentages in the exposed NB + TB conjunctiva were not greater than the control. A similar phenomenon was observed for CD45^+^CD11c^+^CD86^+^ mature dendritic cells (DCs), which were greater in the SB and the combined (SB + NB + TB) groups (Figure 4A–C). The analyses were repeated by comparing the absolute cell number for each population (Supplemental Table S1) and the significance between group differences were the same as those seen in the percentage comparisons. 

### 2.3. Correlations between Conjunctival APCs and KCS Severity Markers

**Figure 5:**
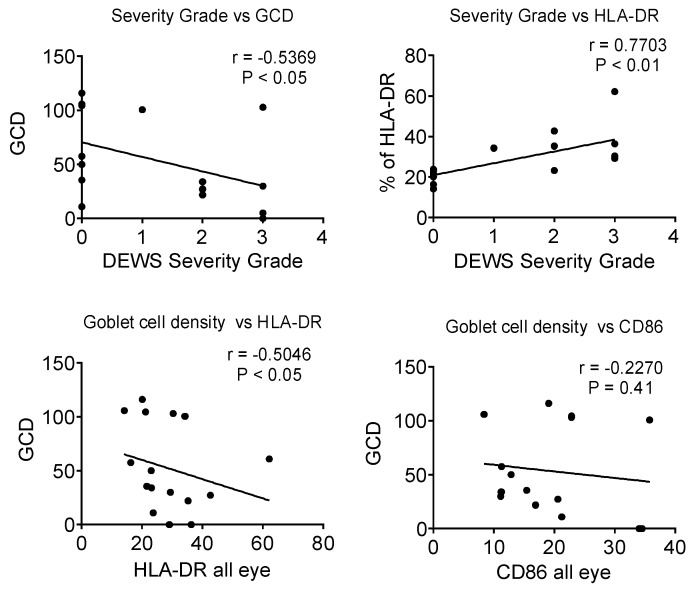
Correlations between clinical severity and goblet cell density (GCD) (**upper left**), clinical severity and percentage of HLA-DR^+^ cells (**upper right**), percentage of HLA-DR^+^ cells and GCD (**lower left**), and percentage of CD86^+^ cells and GCD (**lower right**). Correlations included the control and SS KCS subjects. Spearman *r* and *p* values are provided on graphs.

The Dry Eye Workshop has reported a categorical clinical severity score for KCS based on severity of symptoms, tear break-up time, density of staining of the corneal epithelium with fluorescein and the conjunctival epithelium with lissamine green dye, and other clinical signs [19]. In our study, clinical severity based on this score negatively correlated with GCD (*r* = −0.5, *p* < 0.05) and positively correlated with the percentage of CD45^+^HLA-DR^+^ cells (*r* = 0.77, *p* < 0.01) (Figure 5, top). GCD negatively correlated with percentage of HLA-DR^+^ cells (*r* = −0.54, *p* < 0.05) but not with the percentage of CD86^+^ cells (Figure 5, bottom). None of the correlations were significant if the control subjects were excluded from the analyses.

## 3. Discussion

This study found an increased percentage of bone-marrow-derived cells and APCs in the conjunctiva of patients with SS-associated KCS. A previously reported study had found no difference in percentage of CD45^+^ cells in the conjunctiva between aqueous tear-deficient patients and normal eyes, but the percentage of patients with SS in this cohort was not specified [20]. Our finding of increased CD45^+^ cells in the conjunctiva of SS suggests that there is greater infiltration of bone-marrow-derived cells in this systemic autoimmune condition that has KCS as a defining feature [21]. We also found the percentage of bone-marrow-derived HLA-DR^+^ cells was higher in the SS conjunctiva. These cells could potentially serve as APCs that participate in the adaptive immune response that develops in dry eye [22,23]. There is also a possibility that the increased IFN-γ expression in the SS conjunctiva stimulates more HLA-DR positivity in resident APCs [3].

We evaluated the percentages of HLA-DR^+^ APCs in the nonexposed (superior) and exposed nasal and temporal regions of the bulbar conjunctiva and found a significant difference in percentage between the control and KCS in the superior bulbar conjunctiva. The cause for the greater difference in density of APCs in the superior conjunctiva of SS KCS remains to be determined; it may be because this region is subjected to frequent mechanical trauma from blinking, which promotes leukocyte chemotaxis. This is consistent with the finding of Reinoso and colleagues that the number of T cells and dead epithelial cells are higher in the superior than the inferior conjunctiva in normal eyes [24]. Our finding of increased APC density in the superior conjunctiva may have implications for pathogenesis of KCS and should be confirmed in a separate study. We also found the percentage of APCs expressing the maturation marker CD86 was increased in the SS conjunctiva. CD86 is a costimulatory marker expressed by APCs, which is required for robust antigen-specific activation of T helper cells [25].

We found the number of conjunctival APCs correlated with categorical clinical severity and inversely correlated with the number of goblet cells. It should be noted that while the correlations between goblet cells and disease severity and percentage of HLA-DR^+^ APCs were significant, the correlation coefficients were relatively weak (*r* approximately 0.5) and significance was lost when controls were omitted. This suggests that there are factors other than reduced GCD that contribute to disease severity and HLA-DR expression by APCs. Our findings are consistent with those in the mouse desiccating stress dry eye model that has features resembling SS KCS, including significant goblet cell loss [5,26,27]. Conjunctival APCs are necessary to initiate the adaptive immune response as depletion of phagocytic APCs with clodronate liposomes prevent the T cell-mediated immune response to desiccating environmental stress in this model [28]. It remains to be determined whether reduced conjunctival goblet cell density in SS KCS results in increased APC infiltration/maturation or whether APCs increase in the conjunctiva due to the systemic autoimmune disease and promote the Th1 immune response with increased IFN-γ expression that has been observed in human KCS. The Spdef mouse strain that lacks goblet cells has been found to have an increased number of APCs with greater production of IL-12 in their conjunctiva [8]. The findings of this study suggest that APCs are potential therapeutic targets, and therapies suppressing APC infiltration and activation may have efficacy in SS-associated KCS. A weakness of the study is the small sample size and the lack of a non-SS aqueous-deficient control group with less severe disease. The significance of our findings will have greater strength if they are confirmed in another study with a larger sample size that also includes a non-SS aqueous deficiency (ATD) group.

## 4. Methods

### 4.1. Human Subjects

The study was conducted in accordance with the Declaration of Helsinki, and the Baylor College of Medicine Institutional Review Board approved the protocol and informed consent form prior to study initiation (H-8950, 30 March 2016). Written informed consent was obtained from all participants after explanation of the purpose and possible consequences of the study. Patients with SS were recruited from the multispecialty SS clinic at Baylor College of Medicine (BCM). All SS patients had a complete ocular, oral, and rheumatologic evaluation, including a panel of serum autoantibodies, and met proposed American College of Rheumatology diagnostic criteria for SS [29]. 

Symptom assessment in dry eye (SANDE) and Ocular Surface Disease Index (OSDI) symptom questionnaires, fluorescein tear break-up time (TBUT), Schirmer I test, cornea fluorescein and conjunctival lissamine green dye staining, and tear meniscus height measurement using optical coherence tomography (OCT) were performed as previously described [30,31]. The ocular surface clinical parameters were all measured by the same observer (S.C.P.). Dry Eye Workshop (DEWS) criteria were used to grade clinical severity [19]. 

Control subjects had no eye irritation, a TBUT ≥ 8 s, Schirmer 1 ≥ 10 mm, tear meniscus height ≥ 240 μm, and no meibomian gland disease. Subjects were excluded if they had prior laser assisted in situ keratomilieusis (LASIK) or corneal transplantation surgery, cataract surgery in the past year, punctal occlusion with plugs or cautery, a history of contact lens wear, use of topical medications other than preservative-free artificial tears, or chronic use of systemic medications known to reduce tear production. They were instructed not to instill any tear drops on the day of the evaluation.

### 4.2. Conjunctival Goblet Cell Density

Goblet cell density was measured in impression cytology specimens taken from the temporal bulbar conjunctiva of the left eye. Membranes were fixed and stained by periodic acid Schiff (PAS) reagent as previously described [3,32]. Goblet cells were counted in five representative images taken using image analysis software (Nikon Elements, Garden City, NY, USA), normalized per area, and expressed as GC/mm^2^. GCD was measured in digital images of PAS-stained membranes taken from the temporal bulbar conjunctiva of the left eye.

### 4.3. Flow Cytometry

APCs were obtained from impression cytology of the superior and exposed (nasal + temporal) bulbar conjunctiva of the right eye. Flow cytometry was used to measure the percentage of bone-marrow-derived (CD45) cells that were positive for HLA-DR, CD11c, and CD86 in the conjunctival cells. After the cytology membranes were removed from the eye, they were placed in capped tube containing Roswell Park Memorial Institute (RPMI) media and placed on an oscillating shaker for 1 h. Any remaining cells on the membrane were removed with a cytology brush and were pelleted by centrifugation. Cells were stained with a panel of antibodies including CD45-APC (clone 2D1, eBioscience, Waltham, MA, USA), HLA-DR-FITC (clone L243; Thermofisher, Waltham, MA, USA), CD11c-PE (clone B-ly6, BD Pharmingen, San Diego, CA, USA) and CD86-Pacific blue (clone IT2.2, Biolegend, San Diego, CA, USA), and blue dead Fixable Dead Cell Stain™ (Life Technologies, Grand Island, NY, USA). Negative controls were isotype control antibodies for each color. Flow cytometry was performed with a BD Canto II Benchtop cytometer with BD Diva software version 6.7 (BD Biosciences, San Jose, CA, USA). Cells were initially gated by CD45 vs. live/dead dye followed by two single gates, then gated by HLA-DR followed by CD11c and CD86. A minimum of 100,000 events were acquired. Final data was analyzed with FlowJo software version 10 (Tree Star Inc., Ashland, OR, USA). 

### 4.4. Statistical Analysis

The sample size was calculated using StatMate 2 (GraphPad Software Inc., San Diego, CA, USA) based on pilot studies to have at least 90% power to detect differences with an alpha of 0.05. Based on normality, parametric Student’s *t*-test or nonparametric Mann–Whitney *U* tests were performed for statistical comparisons with an alpha of 0.05 using GraphPad Prism 7.0 software (GraphPad Software Inc.). Spearman correlations between severity and percentages of immune cell markers were calculated. A sample size of eight subjects per group had 90% power of detecting a between-group mean difference of 13% for HLA-DR (α = 0.05).

## Supplementary Materials

Supplementary materials can be found at http://www.mdpi.com/1422-0067/19/9/2760/s1.
