# Supplementary material for: Severity of Sjögren’s Syndrome Keratoconjunctivitis Sicca Increases with Increased Percentage of Conjunctival Antigen-Presenting Cells
Source: Int J Mol Sci. 2018 Sep 14;19(9):2760. doi: 10.3390/ijms19092760 (PMC6165102; doi:10.3390/ijms19092760)
Supplement: Supplementary file 1 [file ijms-19-02760-s001.pdf]

Table S1. Comparison of absolute cell number

	$CD45^+HLA\text{-}DR^+$	$CD45^+CD11c^+HLA\text{-}DR^+$	$CD45^+CD11c^+CD86^+$
Controls	97.6 ± 72.2	80.3 ± 63.7	84.5 ± 63.7
SS KCS	357.9 ± 365.7*	292 ± 303.2*	271.6 ± 333.9†

*P<0.03 vs controls; †P=0.05
